# Spectrofluorimetric Method for Determination of Duloxetine Hydrochloride in Bulk and Pharmaceutical Dosage Forms

**DOI:** 10.4103/0250-474X.44603

**Published:** 2008

**Authors:** S. L. Prabu, S. Shahnawaz, C. Dinesh Kumar, A. Shirwaikar

**Affiliations:** Department of Pharmaceutical Quality Assurance, Manipal College of Pharmaceutical Sciences, Manipal-576 104, India; 1 Department of Pharmacognosy, Manipal College of Pharmaceutical Sciences, Manipal-576 104, India; 2 Department of Pharmaceutics, Manipal College of Pharmaceutical Sciences, Manipal-576 104, India

**Keywords:** Spectrofluorimetry, duloxetine hydrochloride

## Abstract

A simple accurate, sensitive and reproducible spectrofluorimetric method was developed for the analysis of duloxetine hydrochloride in pure and pharmaceutical dosage form. Duloxetine hydrochloride showed strong native fluorescence in 0.05 M acetic acid having excitation at 225 nm and emission at 340 nm. Effect of different solvents were thoroughly investigated. The calibration graph was linear in the range from 0.020 to 0.400 μg/ml. The proposed method was statistically validated and successfully applied for analysis of capsule dosage forms. The limit of detection and limit of quantification were found to be 0.003 μg/ml and 0.010 μg/ml, respectively. The percentage recovery was found to be in the range of 98.71% to 99.17%.

Duloxetine hydrochloride, ((+)-(S)-N-methyl-gamma- (1-naphthyloxy)-2-thiophenepropylamine hydrochloride)^1^, is a potent antidepressant which also acts as a central analgesic^2^. A review of literature revealed that several methods have been reported for the assay of duloxetine^3^–^5^. The aim of the present work was to develop a spectrofluorimetric method that is sensitive, simple, reproducible, rapid and inexpensive.

All fluorescence measurements were done on a Spectrofluorimeter RF-5301 PC (Shimadzu, Japan), with single quartz cell of 1 cm path length. Duloxetine hydrochloride was given as a gift sample from Zydus Cadila, Ahmedabad, India. Methanol, sodium hydroxide and acetic acid were of AR grade, procured form SD Fine Chemicals, Mumbai, India.

A stock solution of duloxetine hydrochloride (500 μg/ml) was prepared. Aliquots of 500 μg/ml solution were suitably diluted with 0.05 M sulphuric acid to give the final concentration in the range of 0.020–0.400 μg/ml. The solution was scanned in the range of 200 to 500 nm against 0.05 M acetic acid as blank, to obtain the excitation and emission wavelength. The excitation and emission wavelength were found to be 225 nm and 340 nm, respectively.

For analysis of duloxetine hydrochloride in solid dosage form, two commercial brands, (Dulojoy, Torrent Pharmaceuticals and Dumore, Lupin Pharmaceuticals), of duloxetine hydrochloride (20 mg strength) were procured from local market. Twenty capsule contents of each brand were weighed and powdered for analysis. The capsule powder, equivalent to 20 mg of duloxetine hydrochloride was weighed accurately, transferred to a clean 100 ml volumetric flask and dissolved in methanol and the final volume was made up. The solution was filtered through Whatmann filter paper No. 40, and conveniently diluted with 0.05 M acetic acid to get the required concentration.

Recovery studies were done at three different levels. The preanalyzed samples were spiked with 80%, 100% and 120% of the standard duloxetine hydrochloride and the mixtures were reanalyzed by the proposed method. The estimation was made in triplicate. Percentage recovery was calculated from the amount of drug found in the solution.

In the present work we discuss the application of spectrofluorimetry to determine the amount of duloxetine hydrochloride in a pharmaceutical sample (capsules). Duloxetine hydrochloride standard preparations were prepared in various mediums like water, dilute acetic acid, 0.1 M NaOH, 0.025 M NaOH, 0.05 M NaOH, methanol, ethanol, acetonitrile, 0.1 M acetic acid, 0.025 M acetic acid and 0.05 M acetic acid.

Duloxetine hydrochloride showed stronger native fluorescence property in 0.05 M acetic acid; hence it was selected as an optimum solvent for spectrofluorimetric analysis. The proposed method for determination of duloxetine hydrochloride in capsule formulation was found to be simple, accurate, economical and rapid. Duloxetine hydrochloride exhibited maximum excitation and emission wavelength at 239 nm and 340 nm, respectively. The linearity was shown in the concentration range of 0.020-0.400 μg/ml (y=752.58×-13.251; correlation coefficient r^2^ = 0.9989). The overlain spectra are shown in fig. 1.

**Fig. 1:**
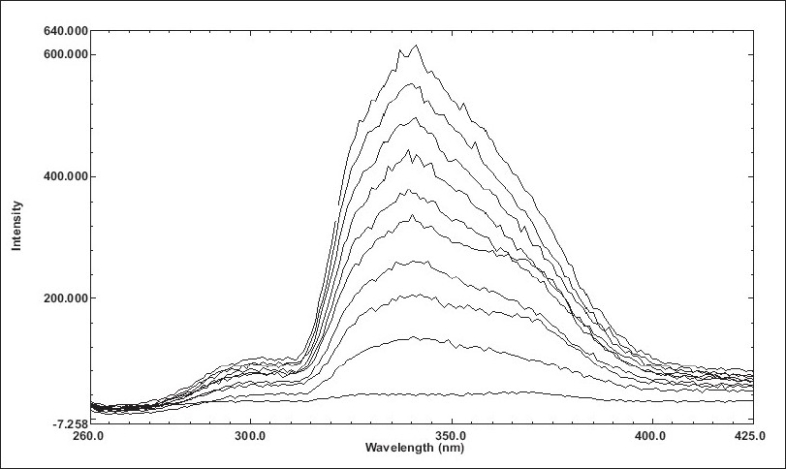
Overlain spectra of duloxetine hydrochloride. Overlain spectra of duloxetine hydrochloride in 0.05 M acetic acid, where, X axis depicts the wavelength and Y axis depicts the intensity.

The results for analysis of commercial formulation are shown in Table 1. The percentage recovery studies revealed that the recovery levels lie between 98.71% and 99.17%. Recovery results demonstrated that the proposed method was unaffected in the presence of formulation excipients and thus highly accurate. Both inter-day as well as intra-day precision carried out, showed that the RSD (relative standard deviation) is less than 2.0 (Table 2). Limit of detection (LOD) and limit of quantification was found to be 3 ng/ml and 10 ng/ml, respectively. Results obtained confirmed ruggedness of the method. The proposed method was valid with respect to linearity, sensitivity, accuracy, reproducibility and precision. The developed method was found to be accurate, precise, reproducible and stable, which indicated that this method can be used for routine quality control of duloxetine hydrochloride in bulk and its solid dosage form.

**TABLE 1:** ANALYSIS OF COMMERCIAL FORMULATION OF DULOXETINE HYDROCHLORIDE

Labeled amount (mg/capsule)	Observed amount (mg/capsule)	% Label claim found±SD
20 (Brand A)	19.88±0.02	99.42±0.08
20 (Brand B)	19.90±0.05	99.50±0.24

The results are mean of three readings (n=3),% Label claim is expressed as mean±standard deviation

**TABLE 2:** INTER AND INTRA-DAY PRECISION FOR DULOXETINE HYDROCHLORIDE ASSAY IN A PHARMACEUTICAL DOSAGE FORM

Concentration of Duloxetine hydrochloride (μg/ml)	Observed concentration of duloxetine hydrochloride			
	
	Intra-day		Inter-day	
	
	Mean (n=5)	RSD%	Mean (n=5)	RSD%
0.100	0.0994	0.76	0.0992	0.71
0.150	0.1472	0.67	0.1481	0.56
0.200	0.1986	0.42	0.1982	0.54

The results mentioned for intra-day and inter-day concentrations are mean of five readings (n=5)
